# The Gender Gap in the Relationship between Metabolic Syndrome and Restrictive Ventilatory Defects

**DOI:** 10.3390/nu16152548

**Published:** 2024-08-03

**Authors:** Ya-Chun Chu, Chi-Chiang Yang, Shaw-Ji Chen, Pei-Ling Cheng, Mei-Chuan Wu, Hsin-Hung Wu, Cheng-Yen Lai

**Affiliations:** 1 Department of Respiratory Therapy Center, Taitung MacKay Memorial Hospital, No. 1, Lane 303, Changsha Street, Taitung City 95054, Taiwan; ivan511084@gmail.com (Y.-C.C.);; 2 Master Program in Biomedicine, College of Science and Engineering, National Taitung University, No. 684, Sec. 1, Zhonghua Rd., Taitung City 95002, Taiwanshawjichen@gmail.com (S.-J.C.); 3 Biomedicine, Agriculture and Food Science Research Center, National Taitung University, No. 684, Sec. 1, Zhonghua Rd., Taitung City 95002, Taiwan; 4 Department of Psychiatry, Taitung MacKay Memorial Hospital, No. 1, Lane 303, Changsha Street, Taitung City 95054, Taiwan; 5 Department of Medicine, MacKay Medical College, No. 46, Section 3, Zhongzheng Rd., Sanzhi District, New Taipei City 25245, Taiwan; 6 Department of Pulmonary Medicine, Taitung MacKay Memorial Hospital, No. 1, Lane 303, Changsha Street, Taitung City 95054, Taiwan

**Keywords:** abdominal obesity, dysglycemia, gender gap, lung function, metabolic syndrome

## Abstract

Background: Given the fundamental physiological differences between the sexes, this study aimed to investigate the effect of metabolic syndrome on ventilatory defects stratified by sex. Methods: We conducted a nationwide, pooled, cross-sectional study. Data from 45,788 participants (men, n = 15,859; women, n = 29,929) aged 30 years or more were obtained from the Taiwan Biobank. Age–sex-adjusted and multivariate logistic regression models were used to estimate the risk of developing impaired pulmonary function (restrictive or obstructive ventilatory defects) in individuals with or without metabolic syndromes. Separate models were also used to estimate the effect of metabolic syndrome scores and the effect of individual metabolic abnormalities on the risk of restrictive ventilatory defects. Results: The overall prevalence of metabolic syndrome was estimated to be 15.9% in Taiwan, much higher in men than in women (18.6% versus 14.4%). A significant association was observed between metabolic syndromes and the risk of restrictive ventilatory defects. The risk of developing a restrictive ventilator defect was 35% higher in participants with metabolic syndromes (odds ratio, 1.35; 95% confidence interval, 1.26–1.45) than in those without metabolic syndromes. Elevated blood pressure and a triglycerides abnormality were important predictors of restrictive ventilator defects. Sex-stratified subgroup analyses of the individual metabolic abnormalities indicated that men with abdominal obesity and women with dysglycemia were more likely to develop restrictive ventilatory defects. Conclusions: Our study’s evidence suggested that metabolic syndromes were important predictors of impaired pulmonary function and an increased risk of developing restrictive ventilatory defects, and its risk increased with increasing numbers of metabolic abnormalities.

## 1. Introduction

Metabolic syndrome (MetS) is a condition that includes a cluster of risk metabolic abnormalities for a variety of diseases, including abdominal obesity (a large waist circumference [WC]), high blood pressure (BP), a high level of fasting plasma glucose (FPG), high level of triglyceride (TG), and low level of high-density lipoprotein cholesterol (HDL-C) levels. The MetS prevalence rates differed considerably from as high as 39.4–57.1% in several Western countries (Finland, Italy, and Mexico) to as low as 3.2–15.5% (Poland, China, and Turkey) [1]. In contrast, MetS is one of the most rapidly increasing chronic conditions in the Taiwanese population. According to the Nutrition and Health Survey in Taiwan, the prevalence rate of MetS has nearly tripled from 11.9% in 1993 to 34.6% in 2020 in the adult population [2]. An important consequence is the increased risk of cardiovascular disease, diabetes, hypertension, and hyperlipidemia in people with MetS, which is two to six times higher than in normal people [3]. Respiratory ventilation disorders can be classified into restrictive and obstructive types based on pulmonary function results, both leading to insufficient lung ventilation. Inadequate pulmonary ventilation would irritate the medulla oblongata to compensate by raising respiratory rates. This situation may lower exercise tolerance or cause shortness of breath and orthopnea. Compensation failure would contribute to chronic hypoxia or muscle atrophy, and long-term respiratory failure could lead to changes in lung structure, increasing the risk of pulmonary hypertension and Cor Pulmonale [4]. Studies have indicated a correlation between smoking and respiratory dysfunction [5,6,7]. The chemical substances in cigarettes, such as nicotine, tar, and carbon monoxide, can overstimulate the macrophages, leading to chronic inflammation of the alveoli and airways. This results in decreased lung function and increased susceptibility to lung infections, promoting the development of lung diseases [8].

Given the fundamental physiological differences between the sexes, women use more movement of the chest and ribs than men during deep breathing, while men mainly apply the diaphragm expansion and abdominal muscles to breathe [9]. The Korean National Health and Nutrition Examination Survey reported that WC, BP, FPG, and HDL-C are independently related to the forced vital capacity of the predicted % (FVC % of predicted) in men, while waist circumference significantly correlated with the FVC predicted (%) in women [10]. In contrast, in a study of 6945 Taiwanese participants, a negative correlation was reported between WC and the FVC % predicted in men younger than 45 years, while no correlation between metabolic abnormality and the FVC % predicted was observed in women [11].

Despite many published studies on the association between MetS and impaired pulmonary function, no consistent evidence is available in the literature regarding sex. We aimed to compare the risk of developing impaired pulmonary function between individuals with or without MetS for each gender using a nationwide, pooled, cross-sectional study design in Taiwan. We hypothesized that sex was an independent predictor of impaired pulmonary functions, and the effects of MetS on impaired pulmonary functions differed by sex among the community-dwelling adult population in Taiwan.

## 2. Methods

### 2.1. Study Design and Data Source

We conducted a nationwide, pooled, cross-sectional study using the data obtained from the Taiwan Biobank. The Taiwan Biobank is a large-scale biomedical research database that intends to recruit 200,000 community-dwelling, healthy volunteers aged 30–79 years residing in Taiwan that consists of genetic and medical information on predominantly individuals who have Han Chinese ancestry [12]. All participants in the Taiwan Biobank provided written informed consent before data collection. The vertical data integration of the collected samples provided data that were accessible to the community-dwelling population in Taiwan. As stipulated by the Human Biobank Management Act, the data of all participants were received as de-identified and anonymous with uniquely encrypted identification numbers in the Taiwan Biobank. As it is a reliable data source, studies using this database have been published in top-tier journals [13,14]. This study has been reviewed and approved by the Human Research Ethics Review Committee of the MacKay Memorial Hospital (21MMHIS351e) on 22 December 2021.

### 2.2. Study Population

The data analysis was initiated on 23 January 2022. Using data released on 28 January 2021, a total of 132,720 adult participants recruited between 10 December 2008 and 30 October 2020 were available for analyses. The exclusion criteria were as follows: (1) participants with missing information on their pulmonary function (n = 50,310) and liver and kidney function abnormalities (n = 47); (2) to ensure the precision of the blood glucose and lipids test results, blood sugar test results with less than 8 h of fasting were excluded (n = 21,854); (3) to maintain homogeneity in the prognosis, we excluded all participants with cancers, respiratory diseases, cardiovascular diseases, and metabolic-related diseases (n = 13,011), and covariates due to incomplete data (n = 1710). A total of 45,788 participants aged 30 years or more were included in the final analysis. Detailed information on the comparison between the baseline characteristics of the participants with missing data and those included in our study is provided in Supplementary Table S1.

### 2.3. Analysis of Covariates

Participants’ residential urbanicity was defined according to Liu et al. [15]. Education levels were divided into college and above (university and graduate school or above), middle school education (junior high and senior high school), and elementary school (elementary school or illiterate). Participants were classified into never smoking, formerly smoking (did not smoke in recent six months), or currently smoking (for more than six months). The habit of drinking was defined as 150 cc per week for 6 months, and former drinking was defined as not drinking for more than 6 months. Monthly exercise habits classified the participants using the metabolic equivalent for the task (MET) as never or seldom doing physical activity, doing light physical activity (monthly physical exercise < 1800 MET), moderate physical activity (monthly physical exercise = 1800–3600 MET), or vigorous physical activity (monthly physical exercise > 3600 MET) [16]. MET is a unit that estimates the amount of energy (kcal/kg/hour) used by an individual during physical activity, and the values were multiplied by 150 min as suggested by the World Health Organization [17].

### 2.4. Definition of Metabolic Abnormalities

According to the modified National Cholesterol Education Program Adult Treatment Panel III (NCEP ATP III), individuals who had at least three of the five metabolic abnormalities were diagnosed as having MetS [18]: (1) abdominal obesity: WC ≥ 90 cm in men and ≥80 cm in women; (2) hypertriglyceridemia: TGs ≥ 150 mg/dL or pharmacotherapy for hyperlipidemia; (3) reduced HDL-C level: HDL-C < 40 mg/dL in men and <50 mg/dL in women; (4) elevated BP: systolic blood pressure ≥ 130 mmHg or diastolic blood pressure ≥ 85 mmHg or pharmacotherapy for hypertension; (5) elevated FPG ≥ 100 mg/dL or pharmacotherapy for glucose-lowering medication.

### 2.5. Pulmonary Function Testing

The pulmonary function was performed by a Lilly-type pneumotach sensor spirometer (HI-801, CHEST M.I., Inc., Tokyo, Japan) with specially trained technicians according to the American Thoracic Society recommendations. All spirometric parameters were repeated three or more times with similar results to meet the criteria for acceptability and reproducibility. The spirometric parameters were compared with the estimated values of the predictive lung function formula, adjusting for race, sex, age, and height, which were used to calculate the FEV1 (forced expiratory volume in one second) predicted %, FVC (forced vital capacity) predicted %, and FEV1 to FVC ratio. Respiratory dysfunction can be classified into an obstructive ventilatory defect (OVD) or a restrictive ventilatory defect (RVD). The pulmonary function test results were classified as normal ventilatory function (FEV1/FVC ≧ 70%), RVD (FEV1/FVC ≧ 70% & FVC < 80% of predicted), or OVD (FEV1/FVC < 70%) [19,20,21,22].

### 2.6. Statistical Analysis

The Chi-squared test for categorial variables and Student’s *t*-test for continuous variables were used to compare group differences. Age–sex-adjusted and multivariate logistic regression models were used to estimate the risk of developing impaired pulmonary function (an RVD or OVD) between individuals with or without MetS, stratified by sex and age. Separate models were also used to estimate the effects of metabolic syndrome scores (MSS) and individual metabolic abnormalities on the risk of RVD. The reported *p* values were based on a two-tailed test, and *p* < 0.05 indicated statistical significance. All data conversions and analyses were performed using SAS for Windows release 9.4 (SAS Institute Inc., Cary, NC, USA).

## 3. Results

### 3.1. Baseline Characteristics

Table 1 compares the basic demographic characteristics (age, obesity status, residential urbanicity, and education level), healthy lifestyle behaviors, family medical history, metabolic abnormalities, and information related to pulmonary impairment between the sexes. Most participants were women (65.4%) aged 30–59 years, resided in an urbanized community, and attended college or graduate school. In general, men had significantly higher incidences of metabolic abnormalities than women, including elevated FPG (29.3% vs. 17.2%), TGs (28.8% vs. 15.2%), and BP (36.8% vs. 20.7%). On the contrary, women had higher incidences of abdominal obesity (28.3% vs. 19.3%) and high-density cholesterol (26.8% vs. 21.6%).

### 3.2. Effects of MetS on Pulmonary Function and Ventilatory Defects

The overall prevalence rate of MetS was estimated to be 15.9% in Taiwan, much higher in men than in women (18.6% versus 14.4%) (Table 1). Of these, 16.7% had an RVD and 13.6% had an OVD. Although a higher proportion of male participants had MetS (18.6% vs. 14.4%) and unhealthy lifestyle behaviors (current smoking, 22.0% vs. 2.9%; current drinking, 13.1% vs. 1.9%) than female participants in this study, the rates of impaired pulmonary function were markedly higher among women for both types of ventilatory defects (RVD, 18.7% vs. 13.0%; OVD, 14.3% vs. 12.3%). In both sex strata, MetS increased the risk of developing impaired pulmonary function (FVC < 80%) (Odds Ratio [OR], 1.33–1.34) similarly to that across all participants (OR, 1.34), which suggested that the outcome was independent of sex. Similarly, the effect of MetS on the risk of developing impaired pulmonary function (FEV1 < 80%, predicted) (OR, 1.19–1.23 vs. 1.22) (Table 2) or an RVD (OR, 1.31–1.36 vs. 1.35) (Table 3) were both independent of sex. On the contrary, MetS did not increase the risk of developing an OVD after covariate adjustment (Table 3). Table 4 shows the subgroup analysis stratified by the MSS scores for the presence of metabolic abnormalities from none (MSS = 0) to five (MSS = 5). In general, both men and women were associated with an increased risk of developing an RVD from anMSS stratification of two to five. Once again, the magnitude of risk for each MSS stratum for each sex was similar to that for all participants, which suggested that the risk of RVDs was independent of sex. Table 5 compares the effects of individual metabolic abnormalities on the risk of RVDs by age and sex categories. In general, both men and women with elevated BP (OR, 1.27–1.46 vs. 1.33) and TG (1.19–1.35 vs. 1.22) abnormalities were more likely to develop an RVD and the risks were independent of both sex and age strata. Conversely, sex-stratified subgroup analyses of the individual metabolic abnormalities indicated that only women with elevated FPG (OR, 1.14–1.38 vs. 1.12) and men with abdominal obesity (OR, 1.24–1.30 vs. 1.07) were more likely to develop an RVD. Although the risk of RVDs was associated with reduced HDL-C by sex (OR, 1.10–1.14) and for all participants (OR, 1.11), the risks of developing RVDs were not statistically different between age strata for men and were only statistically significant in women younger than 55 years (OR, 1.13).

## 4. Discussion

Our study’s evidence suggested that men and women aged 30 years or older with MetS were more likely to suffer from impaired pulmonary function and an increased risk of developing an RVD, and that the risk increased with increasing numbers of metabolic abnormalities. Despite a much higher prevalence of MetS and unhealthy lifestyle (smoking and drinking) behaviors in men than women, similar rates of the increased risk of developing an RVD (31–36%) were found in both sexes, whereas the difference in the risk of developing an OVD was not significant after adjusting for the baseline demographic and lifestyle behaviors. Additionally, elevated BP and TG were both important predictors of an RVD. In contrast, only men with abdominal obesity (or a large WC) and women with dysglycemia (elevated fasting blood glucose) were more likely to develop an RVD. Many studies demonstrated that the risk of developing an RVD was associated with MetS [11,23,24,25,26,27,28]. We observed an increased risk of RVDs with increasing numbers of metabolic abnormalities, which was consistent with the findings reported by other researchers [11,23,29]. However, the results are not consistent in terms of the correlation between MetS and OVDs [30]. Lam et al. classified patients of COPD from I to IV according to the GOLD criteria and found only a significant correlation between MetS and the worst COPD groups [31], which produced similar results to a study on Japanese men [32]. In another study, Lee et al. observed that diabetes is positively associated with RVDs (OR: 2.025, 95% CI: 1.264–3.244), but found no correlation with OVDs (OR: 0.982, 95% CI: 0.634–1.519). They also found that subjects with an RVD had the highest hs-CRP compared to other respiratory dysfunctions [33].

Abdominal obesity is recognized as a major predicting factor for impaired respiratory function, which has been confirmed in many studies [29,34]. Wang et al. found that abdominal obesity lowers FVC and FEV1 values, but it did not affect the FEV1/FVC value [29]. Our study shows that a men’s WC of over 90 cm would decrease ventilatory function, such as FVC, <80% predicted, and FEV1, <80% predicted. In contrast, in a study of 6945 Taiwanese participants, a negative correlation was reported between waist circumference and the FVC % predicted in men younger than 45 years, while no correlation between metabolic abnormality and the FVC % predicted was observed in women [11]. On the other hand, the Korean National Health and Nutrition Examination Survey in 2007 observed that WC, BP, FPG, and HDL-C independently related to the FVC % predicted in men, while WC significantly correlated with the FVC % predicted in women [10]. Obesity in men presents as the deposit of fat in the chest, abdomen, and visceral organs, known as central obesity. In women, fat is usually stored in the buttocks, thighs, hips, and subcutaneous tissue, known as peripheral obesity [35]. Accumulated fat in the mediastinum and abdomen decreases the compliance of the chest wall, causing breathing pattern changes. Intra-abdominal pressure and intrapleural pressure slightly increase in people with obesity, which decreases the differential pressure from the atmosphere to the chest, leading to a ventilatory flow limit. In addition, the downward movement of the diaphragm and expansion of the chest are restricted by adipose tissue in the abdomen, decreasing the expiratory reserved volume and functional residual capacity, increasing labored breathing, and lowering ventilatory efficiency [19,35]. However, it is still unclear whether obesity lowers the compliance of the lungs, chest, or both [36]. Further studies are needed to confirm the association.

MetS is a chronic inflammatory state that triggers an immune response and the proliferation of inflammatory substances which reduce pulmonary function. In our study, we found that men with abdominal obesity (or a large WC) and women with dysglycemia (elevated FPG) had a higher risk of respiratory ventilation defects (RVDs) than those without these metabolic abnormalities. This indicated a sex-specific association between abnormal fasting blood glucose and the secretion of inflammatory substances. Kawamoto et al. reported a significant sex-specific correlation between fasting blood glucose and hsCRP, and women had a stronger correlation than men [37]. The elevated hs-CRP difference showed that women with high blood glucose will have a higher risk of cardiovascular disease than men in the future [38]. Likewise, adipocyte accumulation due to obesity secretes adipocytokines, such as Interleukin-6, TNF-α, interferon-γ, and leptin, stimulates bronchial smooth muscle contraction, and induces macrophages to move into the alveoli, provoking chronic inflammation damage to the alveoli and airway and increasing respiratory symptoms [39].

## 5. Limitation

There are some limitations to be considered while interpreting our study results. Firstly, we used spirometry to measure respiratory function instead of more complex and costly methods like plethysmography, which means we lack data on total lung capacity and residual volume, potentially introducing residual confounding. However, trained personnel using spirometry are sufficient for the early screening of respiratory function in the community. Secondly, limitations include the predominance of participants of Han ethnicity from Taiwan, which may limit the generalizability of our findings to other ethnic groups. We suggest conducting similar studies in diverse populations to improve the generalizability. Voluntary participation may introduce selection bias and limit community diversity. To mitigate this bias, future studies could use Propensity Score Matching (PSM) to create comparable groups based on observed variables, thereby improving the accuracy of results. Moreover, reliance on self-reported lifestyle data may introduce memory and reporting biases. Collecting data at multiple time points can adjust for variability and improve reliability, while comparing it with follow-up data from the Taiwan Biobank can further enhance accuracy. Thirdly, there are other potential factors that could affect respiratory function which we did not address in this study. Occupational exposures such as dust particles, chemicals, and animal droppings as airborne particulates may also lead to respiratory abnormalities over prolonged exposure, but relevant data were lacking in this study. Additionally, the absence of data on inflammatory markers like hs-CRP limits our ability to conduct thorough research into the relationship between inflammation and respiratory function impairment. Moreover, the lack of respiratory symptoms related to ventilatory defects, such as coughing and dyspnea, restricts our assessment of the association between MetS and respiratory symptoms. Lastly, our study is cross-sectional and lacks the consideration of temporal interference factors. Future longitudinal research is essential to establish causal relationships and provide deeper insights into the dynamics between the variables studied.

## 6. Conclusions

Despite the limitations, our study’s evidence suggested that metabolic syndromes were important predictors of impaired pulmonary function and an increased risk of developing restrictive ventilatory defects, and the risk increased with increasing numbers of metabolic abnormalities. If gender-specific prevention strategies are to be be tailored for individuals at an increased risk of impairment pulmonary function, further studies are required to determine the metabolic effects of medications as well as modifiable lifestyle behaviors on metabolic abnormalities.

## Figures and Tables

**Table 1:** Baseline characteristics of total participants stratified by sex (n = 45,788).

Independent Variables	Men	Women	*p*-Value
Participants, n (%)	15,859 (34.6)	29,929 (65.4)	<0.001
Age, mean (SD), y	48.8 (11.3)	49.3 (10.6)	<0.001
Age, n (%)			
30–39 y	4198 (26.5)	6893 (23.0)	<0.001
40–49 y	4053 (25.6)	7618 (25.5)	
50–59 y	4123 (26.0)	9513 (31.8)	
60–69 y	3396 (21.4)	5828 (19.5)	
≥70 y	89 (0.6)	77 (0.3)	
Residential urbanicity, n (%)			
Urban	9329 (58.8)	18,002 (60.2)	0.017
Suburban	5695 (35.9)	10,450 (34.9)	
Rural	835 (5.3)	1477 (4.9)	
Education level, n (%)			
College or graduate School	10,840 (68.4)	16,443 (54.9)	<0.001
High school	4658 (29.4)	11,842 (39.6)	
None or elementary school	361 (2.3)	1644 (5.5)	
Smoking experience, n (%)			
Never smoked	8797 (55.5)	28,305 (94.6)	<0.001
Formerly smoked	3563 (22.5)	747 (2.5)	
Currently smokes	3499 (22.1)	877 (2.9)	
Drinking habits, n (%)			
Never drank	13,005 (82.0)	29,132 (97.3)	<0.001
Formerly drank	774 (4.9)	235 (0.8)	
Currently drinks	2080 (13.1)	562 (1.9)	
Monthly exercise habits, n (%)			
Never or seldom physical activity	9428 (59.5)	18,266 (61.0)	<0.001
Light physical activity	646 (4.1)	1740 (5.8)	
Moderate physical activity	1696 (10.7)	3756 (12.6)	
Vigorous physical activity	4089 (25.8)	6167 (20.6)	
BMI, mean (SD)	25.2 (3.5)	23.5 (3.7)	<0.001
Obese status (BMI ≧ 27), n(%)	4109 (25.9)	4549 (15.2)	<0.001
Body fat rate, n (%)			
Family medical history *	22.9 (5.3)	31.8 (6.3)	<0.001
Asthma	1067 (6.7)	2541 (8.5)	<0.001
Emphysema or Chronic bronchitis	337 (2.1)	828 (2.8)	<0.001
Cardiovascular disease	5383 (33.9)	11,262 (37.6)	<0.001
Diabetes	5081 (32.0)	11,109 (37.1)	<0.001
Metabolic syndrome, n (%)	2955 (18.6)	4304 (14.4)	<0.001
Metabolic abnormalities, n (%)			
Fasting glucose ≧ 100 mg/dL	4642 (29.3)	5143 (17.2)	<0.001
TGs ≧ 150 mg/dL	4559 (28.8)	4647 (15.5)	<0.001
HDL-C < 40 mg/dL in men or HDL-C < 50 mg/dL in women	3421 (21.6)	8005 (26.8)	<0.001
SBP ≧ 130 mmHg or DBP ≧ 85 mmHg,	5834 (36.8)	6180 (20.7)	<0.001
WC ≧ 90 cm in men or WC ≧ 80 cm in women	3061 (19.3)	8468 (28.3)	<0.001
Lung function parameters			
FEV1, mean (SD), L	2.9 (0.8)	2.0 (0.6)	<0.001
FVC, mean (SD), L	3.6 (0.7)	2.4 (0.5)	<0.001
FEV1, % predicted	89.1 (22.0)	83.8 (22.5)	<0.001
FVC, % predicted	94.2 (15.2)	91.0 (16.8)	<0.001
FEV1/FVC ratio	95.2 (20.8)	92.5 (20.7)	<0.001
Lung function parameters, n (%)			
FEV1 < 80% predicted	4084 (25.8)	10,667 (35.6)	<0.001
FVC < 80% predicted	2348 (14.8)	6674 (22.3)	<0.001
Lung function diagnosis, n (%)			
Normal ventilatory function	11,855 (74.8)	20,025 (66.9)	<0.001
Restrictive ventilatory defect	2058 (13.0)	5610 (18.7)	
Obstructive ventilatory defect	1946 (12.3)	4294 (14.3)	

SD, standard deviation; WC, waist circumference; BMI, body mass index; SBP, systolic blood pressure; DBP, diastolic blood pressure; TGs, triglycerides; HDL-C, high-density lipoprotein cholesterol; FVC, forced volume capacity; FEV1, force expiratory volume in one second; *p*-value, χ^2^ test or Student’s *t*-test. * Family medical histories of the participant’s second degree of blood relatives were included in the family medical history. Cardiovascular disease consisted of vascular heart disease, coronary heart disease, arrhythmia, cardiomyopathy, congenital heart disease, hyperlipidemia, hypertension, and stroke.

**Table 2:** The effects of metabolic syndrome on the risk of impaired pulmonary function by sex.

	FVC < 80%, Predicted				FEV1 < 80%, Predicted			
Independent Variables	Age–Sex-Adjusted OR, (95% CI)		Multivariable-Adjusted OR, (95% CI)		Age–Sex-Adjusted OR, (95% CI)		Multivariable-Adjusted OR, (95% CI)	
All participants	1.20 (1.13–1.27) ^c^	***	1.34 (1.25–1.44) ^d^	***	1.05 (1.00–1.11) ^c^		1.22 (1.15–1.30) ^d^	***
Men	1.37 (1.23–1.52) ^b^	***	1.33 (1.18–1.50) ^a^	***	1.18 (1.08–1.29) ^b^	***	1.23 (1.11–1.36) ^a^	***
Women	1.13 (1.05–1.22) ^b^	**	1.34 (1.23–1.46) ^e^	***	0.99 (0.92–1.06) ^b^		1.19 (1.10–1.29) ^e^	***

OR, odds ratio; CI, confidence interval; FVC, forced volume capacity; FEV1, force expiratory volume in one second; *p* < 0.01 **, *p* < 0.001 *** (*p*-value). a. Odds ratio adjusted for age, smoking experience, drinking habits, monthly exercise habits, education level, residential urbanicity, BMI, body fat rate, and family medical history (asthma, emphysema or chronic bronchitis, cardiovascular diseases, diabetes). b. Odds ratio adjusted for only age. c. Odds ratio adjusted for only sex and age. d. Odds ratio adjusted for all variables in footnote a and sex. e. Odds ratio adjusted for all variables in footnote a and menopausal status.

**Table 3:** The effects of metabolic syndrome on the risk of ventilatory defects by sex.

	Restrictive Ventilatory Defect				Obstructive Ventilatory Defect		
Independent Variables	Age–Sex-Adjusted OR, (95% CI)		Multivariable-Adjusted OR, (95% CI)		Age–Sex-Adjusted OR, (95% CI)		Multivariable-Adjusted OR, (95% CI)
All participants	1.27 (1.19–1.35) ^c^	***	1.35 (1.26–1.45) ^d^	***	0.86 (0.79–0.93) ^c^	***	0.97 (0.89–1.06) ^d^
Men	1.44 (1.29–1.61) ^b^	***	1.31 (1.16–1.49) ^a^	***	0.92 (0.81–1.04) ^b^		1.05 (0.91–1.21) ^a^
Women	1.20 (1.11–1.30) ^b^	***	1.36 (1.24–1.48) ^e^	***	0.82 (0.74–0.91) ^b^	**	0.92 (0.82–1.03) ^e^

OR, odds ratio; CI, confidence interval; restrictive ventilatory defect: FEV1/FVC ≧ 70% and FVC, predicted% < 80%; obstructive ventilatory defect: FEV1/FVC < 70%; *p* < 0.01 **, *p* < 0.001 *** (*p*-value). a. Odds ratio adjusted for age, smoking experience, drinking habits, monthly exercise habits, education level, residential urbanicity, BMI, body fat rate, and family medical history (asthma, emphysema or chronic bronchitis, cardiovascular diseases, diabetes). b. Odds ratio adjusted for only age. c. Odds ratio adjusted for only sex and age. d. Odds ratio adjusted for all variables in footnote a and sex. e. Odds ratio adjusted for all variables in footnote a and menopausal status.

**Table 4:** The effects of metabolic syndrome scores on the risk of restrictive ventilatory defects by sex.

OR, (95% CI)	Non-Metabolic Syndrome			Metabolic Syndrome		
	MSS = 0	MSS = 1	MSS = 2	MSS = 3	MSS = 4	MSS = 5
Multivariable-adjusted ^b^	1	1.06 (0.99–1.13)	1.21 (1.11–1.31) ***	1.41 (1.28–1.56) ***	1.51 (1.33–1.72) ***	2.40 (1.94–2.97) ***
Men ^a^	1	1.15 (1.00–1.32) *	1.34 (1.16–1.56) ***	1.52 (1.27–1.81) ***	1.58 (1.26–1.99) ***	2.75 (1.87–4.04) ***
Women ^c^	1	1.04 (0.96–1.13)	1.16 (1.06–1.28) **	1.38 (1.23–1.55) ***	1.49 (1.27–1.74) ***	2.26 (1.75–2.91) ***

OR, Odds ratio; CI, confidence interval; MSS, metabolic syndrome score; *p* < 0.05 *, *p* < 0.01 **, *p* < 0.001 *** (*p*-value). a. Odds ratio adjusted for age, smoking experience, drinking habits, monthly exercise habits, education level, residential urbanicity, BMI, body fat rate, and family medical history (asthma, emphysema or chronic bronchitis, cardiovascular diseases, diabetes). b. Odds ratio adjusted for all variables in footnote a and sex. c. Odds ratio adjusted for all variables in footnote a and menopausal status.

**Table 5:** The effects of each metabolic component on the risk of restrictive ventilatory defects by sex and age.

Independent Variables OR, (95% CI)						
	Whole Population		Men		Women	
Elevated BP ^f^	1.33 (1.25–1.41) ^c^	***	1.27 (1.14–1.40) ^b^	***	1.36 (1.26–1.46) ^e^	***
<55 years old			1.31 (1.13–1.52) ^a^	***	1.32 (1.16–1.49) ^d^	***
>=55 years old			1.40 (1.22–1.61) ^a^	***	1.46 (1.34–1.60) ^d^	***
Elevated FPG ^g^	1.12 (1.05–1.20) ^c^	***	1.05 (0.94–1.16) ^b^		1.15 (1.07–1.25) ^e^	***
<55 years old			1.07 (0.91–1.26) ^a^		1.38 (1.21–1.57) ^d^	***
>=55 years old			1.21 (1.05–1.39) ^a^	**	1.14 (1.04–1.26) ^d^	**
Reduced HDL-C ^h^	1.11 (1.04–1.18) ^c^	***	1.14 (1.02–1.28) ^b^	*	1.10 (1.03–1.18) ^e^	**
<55 years old			1.15 (0.98–1.36) ^a^		1.13 (1.02–1.24) ^d^	*
>=55 years old			1.13 (0.96–1.33) ^a^		1.07 (0.97–1.18) ^d^	
Elevated TGs ^i^	1.22 (1.14–1.30) ^c^	***	1.27 (1.15–1.42) ^b^	***	1.19 (1.09–1.29) ^e^	***
<55 years old			1.35 (1.16–1.56) ^a^	***	1.22 (1.07–1.38) ^d^	***
>=55 years old			1.25 (1.07–1.46) ^a^	**	1.20 (1.08–1.34) ^d^	***
Abdominal obesity ^j^	1.07 (1.00–1.16) ^c^	*	1.26 (1.08–1.46) ^b^	**	1.03 (0.95–1.13) ^e^	
<55 years old			1.30 (1.04–1.61) ^a^	*	1.09 (0.96–1.24) ^d^	
>=55 years old			1.24 (1.00–1.53) ^a^	*	1.00 (0.89–1.12) ^d^	

*p* < 0.05 *, *p* < 0.01 **, *p* < 0.001 *** (*p*-value) a. Odds ratio adjusted for smoking experience, drinking habits, monthly exercise habits, education level, residential urbanicity, BMI, body fat rate, family medical history (asthma, emphysema or chronic bronchitis, cardiovascular diseases, diabetes). b. Odds ratio adjusted for all variables in footnote a and age. c. Odds ratio adjusted for all variables in footnote a, sex and age. d. Odds ratio adjusted for all variables in footnote a and menopausal status. e. Odds ratio adjusted for all variables in footnote a, age, and menopausal status. f. Elevated BP: systolic blood pressure ≧ 130 mmHg or Diastolic blood pressure ≧ 85 mmHg. g. Elevated FPG: fasting plasma glucose ≧ 100 mg/dL. h. Reduced HDL-C: high-density lipoprotein cholesterol < 40 mg/dL in men or <50 mg/dL in women. i. Elevated TGs: triglycerides ≧ 150 mg/dL. j. Abdominal obesity: waist circumference ≧ 90 cm in men or ≧80 cm in women.

## Data Availability

The data that support the findings of this study are available from the Taiwan Biobank, but restrictions apply to the availability of these data, which were used under license for this study. Data are, however, available from the authors upon reasonable request with the permission of Taiwan Biobank.

## Supplementary Materials

The following supporting information can be downloaded at: https://www.mdpi.com/article/10.3390/nu16152548/s1, Table S1: Comparison of baseline characteristics between participants with missing data * and those included in the study.
