# Supplementary material for: The Gender Gap in the Relationship between Metabolic Syndrome and Restrictive Ventilatory Defects
Source: Nutrients. 2024 Aug 3;16(15):2548. doi: 10.3390/nu16152548 (PMC11314350; doi:10.3390/nu16152548)
Supplement: Supplementary file 1 [file nutrients-16-02548-s001.zip › nutrients-3112570-supplementary.pdf]

Supplement Table

Table S1. Comparison of baseline characteristics between participants with missing data* and those included in the study.

Independent Variables	Included participants (n=45,788)	Missing data	Missing Numbers
Man, n (%)	15,859(34.6)	17,243(34.2)	50,357
Age, y	49.1(10.8)	49.5(11.1)	50,357
Laboratory parameters			
HbA1C	5.7(0.7)	5.8(0.8)	50,255
Fasting glucose, mg/dL	95.9(19.8)	96.2(21.6)	50,276
TGs, mg/dL	113.2(89.2)	115.9(98.3)	50,275
HDL-C, mg/dL	54.8(13.3)	54.7(13.5)	50,275
LDL-C, mg/dL	121.6(31.8)	121.0(32.0)	50,275
TC, mg/dL	195.9(35.7)	195.7(39.2)	50,275
Physical examination			
BMI	24.1(3.7)	24.3(3.9)	50,276
Body fat rate, %	28.7(7.3)	28.9(7.5)	48,576
Height, cm	162.0(8.3)	161.7(8.3)	50,280
Weight, cm	63.5 (12.6)	63.7(12.9)	50,276
WC, cm	82.7(10.0)	83.3(10.5)	50,289
SBP, mmHg	117.8(17.8)	118.9(18.1)	50,356
DBP, mmHg	72.7(11.0)	73.0(11.0)	50,357

Values are means with SD in parentheses (continuous variables).

* Missing data include pulmonary function and metabolic abnormalities.

SD, standard deviation; WC, waist circumference; BMI, body mass index; SBP, systolic blood pressure; DBP, diastolic blood pressure; HbA1C, hemoglobin A1c; TGs, triglycerides; HDL-C, high-density lipoprotein Cholesterol; LDL-C, low-density lipoprotein Cholesterol; TC, total cholesterol.
